# Synthetic Cannabinoids: Metabolism, Biomarkers of Exposure and Advanced Analytical Strategies in Forensic and Clinical Toxicology

**DOI:** 10.3390/toxics14100898

**Published:** 2026-10-10

**Authors:** Telma M. Gomes, Paula Guedes de Pinho, Ivone Nunes, José S. Câmara

**Affiliations:** 1 CQM—Centro de Química da Madeira, Universidade da Madeira, Caminho da Penteada, 9020-105 Funchal, Portugal; 2 UCIBIO—Applied Molecular Biosciences Unit, Laboratory of Toxicology, Faculty of Pharmacy, University of Porto, 4050-313 Porto, Portugal; 3 Associate Laboratory i4HB—Institute for Health and Bioeconomy, University of Porto, 4050-313 Porto, Portugal; 4 Centro de Tratamento de Adições, Departamento de Saúde Mental, Hospital dos Marmeleiros, Serviço Regional de Saúde da Região Autónoma da Madeira (SESARAM), EPE, Estrada dos Marmeleiros, 9054-535 Funchal, Portugal; 5 Departamento de Química, Faculdade de Ciências Exatas e Engenharia, Universidade da Madeira, Campus da Penteada, 9020-105 Funchal, Portugal

**Keywords:** new psychoactive substances, synthetic cannabinoids, metabolomics, in vitro/in vivo models, biomarkers of exposure

## Abstract

In the last twenty years, synthetic cannabinoid receptor agonists (SCRAs), also frequently known as synthetic cannabinoids, have grown into one of the leading and structurally diverse types of new psychoactive substances. This has brought about major trouble for clinical and forensic work. Even though there have been significant advances in the field, there remain important gaps in the understanding of the mechanism of action, biotransformation pathways, etc. Numerous studies indicate that optimized chromatographic–mass spectrometric technologies are vital for the sensitive and specific detection of SCRAs and their metabolites from complex biological matrices. Studies that employ rigorous accreditation-level protocols highlight the need for a method validation process for the reliable detection of compounds. Meanwhile, scientifically controlled studies involving humans provide information on the connection between a compound and its metabolites with the clinical effects. In parallel, metabolomic investigations suggest that SCRA metabolism is closely tied to cellular dysregulation and adverse toxicological outcomes, indicating that biotransformation can generate reactive intermediates and contribute directly to toxicity. Overall, by combining metabolite profiling, emerging biomarkers and structure-informed toxicological interpretation, the literature points toward a more integrated and predictive framework to guide future analytical strategies and support evidence-based harm reduction measures.

## 1. Introduction

The past two decades have witnessed a remarkable increase in both the frequency and structural diversity of new psychoactive substances (NPSs). Among this heterogeneous group, synthetic cannabinoid receptor agonists (SCRAs), commonly referred to as synthetic cannabinoids, represent one of the largest and most extensively documented subclasses. Unlike Δ^9^-tetrahydrocannabinol (Δ^9^-THC), the main psychoactive constituent of cannabis, SCRAs are structurally diverse synthetic compounds designed to mimic its pharmacological effects, often with substantially greater receptor affinity and potency. Their high potency and ready availability and the continuous emergence of novel analogs have established SCRAs as one of the most prevalent groups of NPSs encountered in forensic and toxicological practice. First identified in the USA, Russia, and Canada, SCRAs began to appear in Europe around 2005 [1,2]. More recently, semi-synthetic cannabinoids, such as hexahydrocannabinol (HHC), have also emerged; however, owing to differences in their synthetic origin and chemical structure relative to illicit SCRAs, these compounds fall outside the scope of the present review. The combination of high potency, widespread availability, and low cost has contributed to the disproportionate use of SCRAs among vulnerable populations, including incarcerated and homeless individuals, further compounding the public health challenges associated with these substances [2].

The rapid emergence of new psychoactive substances (NPSs), driven by extensive structural modifications to the core scaffolds of parent molecules, has led to a proliferation of chemical variants within remarkably short time frames. This structural diversity significantly hampers comprehensive investigations into their pharmacological properties, toxicity profiles, and biotransformation pathways. The identification of these substances and their metabolites in biological matrices is further complicated by their rapid and extensive metabolic conversion. Consequently, innovative analytical strategies are being developed to address these challenges. Among these, metabolomic approaches have played an important role in elucidating the alteration of metabolic pathways resulting from cellular exposure to these molecules. They are also valuable for confirming the biotransformation characteristics of NPSs, including the detailed chemical structures of phase I and phase II metabolites, as well as the associated dysregulation of metabolic pathways [3]. Such insights are crucial for: (i) the development of clinical and forensic diagnostic assays based on biological fluids, where metabolites rather than parent compounds serve as primary analytes; (ii) elucidating and assessing the mechanistic basis of acute and chronic toxic effects induced by NPSs; (iii) enabling rapid toxicological risk assessment; and (iv) identifying unique biomarkers for the reliable confirmation of drug exposure in clinical and forensic toxicology settings [3].

A variety of in vitro and in vivo models, including pooled human liver microsomes (pHLMs) and rat urine, are commonly employed in drug metabolism studies [4]. Among these, the pHLM model is widely regarded as a gold-standard in vitro system due to its complement of drug-metabolizing enzymes and ease of accessibility. It is extensively used for the identification of both phase I and phase II metabolites. Importantly, most drugs undergo metabolism through cytochrome P450 (CYP)-mediated oxidation and uridine 5′-diphospho-glucuronosyltransferase (UGT)-mediated glucuronidation [5,6]. In addition, incubation with human hepatocytes provides a robust in vitro alternative to animal models, generating comprehensive metabolic profiles with relevant phase I and II metabolites at concentrations closely mirroring those observed in vivo [7]. Metabolomic investigations are pivotal for elucidating the cellular metabolic pathways disrupted following exposure to SCRAs in both in vitro systems and human biological matrices. Typically, such studies employ two principal analytical platforms—(i) mass spectrometry (MS) coupled with gas or liquid chromatography and (ii) nuclear magnetic resonance (NMR) spectroscopy—each offering complementary insights into metabolic alterations. Given this context, the objective of the present narrative review is to provide a comprehensive and critical synthesis of the current knowledge on SCRAs, integrating analytical, metabolic, and toxicological perspectives. Specifically, this review aims to (i) characterize the structural diversity of SCRAs and the analytical challenges it poses for their sensitive and selective detection in biological matrices; (ii) critically appraise the chromatographic–mass spectrometric platforms and validation workflows currently employed for the identification and quantification of SCRAs and their metabolites, with particular attention to accreditation-level standards relevant to forensic and clinical practice; (iii) summarize the biotransformation pathways of representative SCRAs, highlighting major and minor metabolites of toxicological and forensic relevance; and (iv) examine the relationship between SC metabolism and cellular toxicity, with an emphasis on the role of reactive metabolic intermediates in mediating adverse effects. By bringing together these strands of evidence, this review seeks to outline a more integrated and predictive framework to guide future analytical strategies and support evidence-based harm reduction approaches.

## 2. Literature Search Strategy

Extensive literature research was carried out to find relevant studies on metabolism, biotransformation and analysis of synthetic cannabinoids. A search was conducted using PubMed, Google Scholar, and ScienceDirect online databases. Search terms included “synthetic cannabinoids” together with such keywords as “metabolism,” “biotransformation,” “metabolomics,” “metabolic profiling,” and “toxicology”.

The inclusion criteria comprised the following:

(i) Articles published in English in peer-reviewed journals;
(ii) Studies that specifically address synthetic cannabinoids;
(iii) Studies that investigated metabolism, biotransformation, metabolic profiling, biomarkers of exposure, toxicological aspects or analytical detection methods;
(iv) Studies that involved human biological samples, relevant in vitro models or analytical methods applicable to the investigation of synthetic cannabinoids.

Studies were excluded if they (i) were not directly related to synthetic cannabinoids; (ii) focused exclusively on other classes of new psychoactive substances without providing specific information on synthetic cannabinoids; (iii) did not provide relevant metabolic, toxicological or analytical information; or (iv) were duplicate publications. Additional references were found by the manual bibliography screening of selected articles.

The literature was assessed for its scientific relevance, methodological quality, and contributions to knowledge on the metabolism, biomarkers, and toxicological significance of synthetic cannabinoids. Extracted information from the selected studies includes the investigated compound, biological sample type, used analytical methodology, detected metabolites, metabolic pathways, and main findings.

## 3. New Psychoactive Substances—Synthetic Cannabinoids

**Figure 1:**
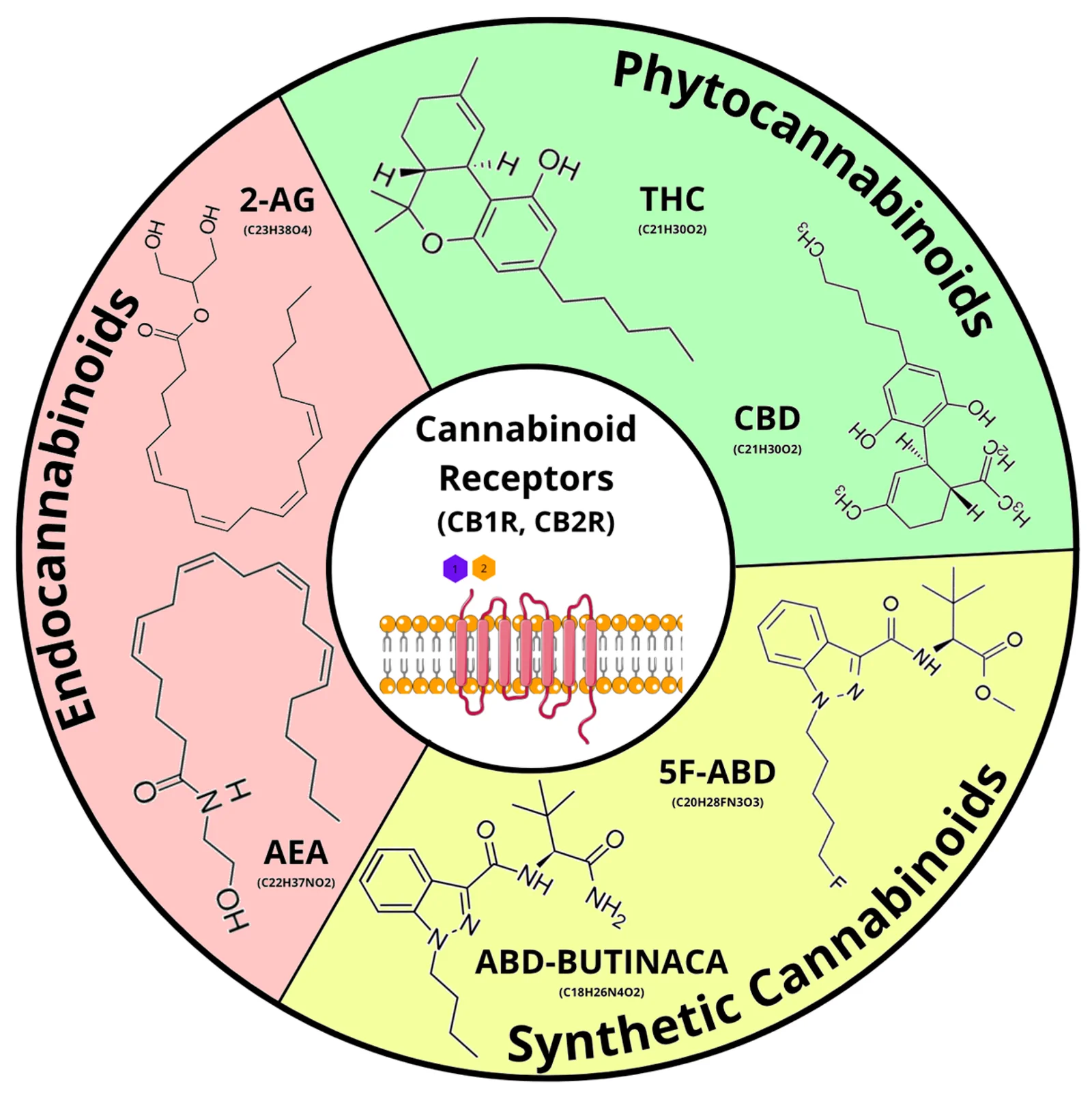
Compounds that can bind to cannabinoid receptors: endocannabinoids, phytocannabinoids and synthetic cannabinoids. Compounds that can bind to cannabinoid receptors: endocannabinoids, phytocannabinoids and synthetic cannabinoids. Created using Canva (https://www.canva.com/) (Canva Pty Ltd., Sydney, Australia). The purple and orange hexagons represent CB1R and CB2R, respectively.

Cannabinoids are lipid-soluble, non-polar compounds, typically composed of 20–26 carbons atoms, and they comprise a diverse group of molecules capable of binding to cannabinoid receptors in the human body [8]. These compounds arise from different sources, may be endogenous, and are synthesized naturally within the organism; they can be present in the cannabis plant (phytocannabinoids) or produced in the laboratory (synthetic analogs, named synthetic cannabinoids) [9], as shown in Figure 1.

Of the major phytocannabinoids found in *Cannabis sativa*, cannabidiol (CBD) is of particular interest, alongside Δ^9^-tetrahydrocannabinol (Δ^9^-THC). Unlike Δ^9^-THC, CBD does not produce the characteristic intoxicating effects associated with cannabis, instead exhibiting a distinct and complex pharmacological profile [10]. This profile differs markedly from that of SCRAs, which bind to and activate CB1R and CB2R, and which may exhibit a higher affinity for CB1R than Δ^9^-THC [11]. Therefore, considering CBD alongside Δ^9^-THC provides the relevant context for distinguishing naturally occurring phytocannabinoids from potent synthetic agonists encountered as new psychoactive substances.

The initial motivation for synthesizing cannabinoids was to replicate the therapeutic effects of the phytocannabinoid Δ^9^-tetrahydrocannabinol (THC) used in managing conditions such as cancer, chronic pain, anorexia, muscle spasms, and glaucoma while minimizing or eliminating its psychoactive properties [2,9,12]. To achieve this, researchers developed a range of THC analogs in the laboratory, giving rise to the class of synthetic cannabinoids. However, rather than reducing psychoactivity, many of these analogs exhibited significantly greater potency than THC, often amplifying its psychoactive and toxicological effects. Whereas THC is a partial agonist of cannabinoid receptor type 1 (CB1R), numerous synthetic cannabinoids act as full agonists, exhibiting up to threefold greater binding affinity for CB1R [2,9].

**Figure 2:**
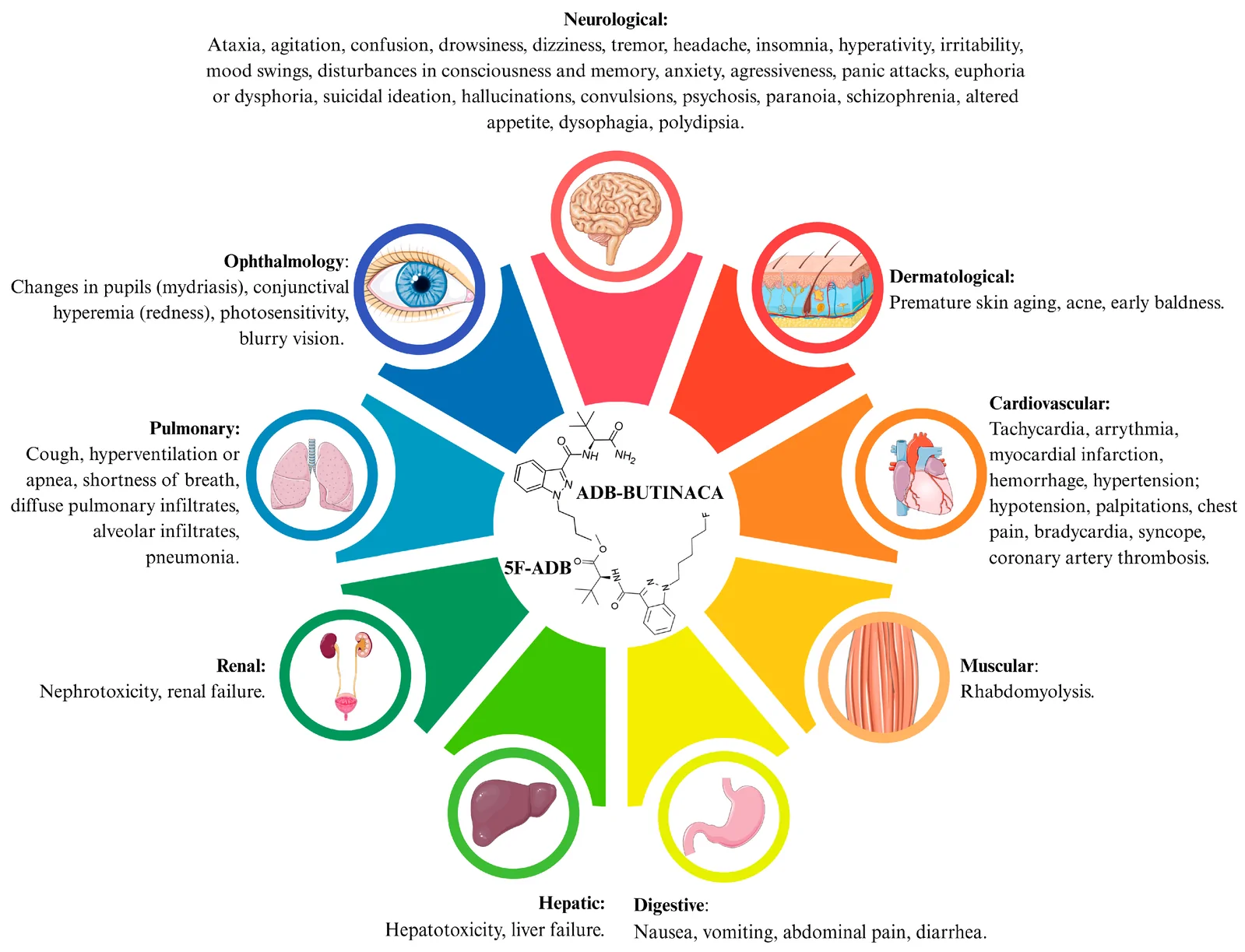
Most common adverse effects provoked by SC intoxication. Adapted from Roque-Bravo et al. 2023 [14]. 2023, Annu. Rev. Pharmacol. Toxicol. Created using Canva (https://www.canva.com/).

Nowadays, these synthetic substances are called “spice” (in Europe) and are sold as “legal” substitutes for cannabis, and this characteristic of the “full agonism activity” of SCRAs has revealed their high potency, resulting in a range of different behavioral and physiological effects, such as relaxation, euphoria, distorted perception of time, hallucinations, lethargy, dry mouth, paranoia, agitation, confusion, anxiety, bloodshot eyes, nausea, fear, tachycardia, vomiting, and, in severe intoxication, rapid loss of consciousness, hyperemesis, delirium, psychosis, respiratory depression, violent behavior, cognitive impairment, stroke, seizures, cardiac complications, acute renal failure, acute liver injury, and even death [2,8,13]. The adverse effects and organs affected are shown in Figure 2.

Currently, there is no specific antidote for acute intoxication with SCRAs. Management is therefore primarily supportive and symptom oriented. Initial care should include monitoring vital signs and cardiorespiratory status, with the administration of intravenous fluids and other supportive measures as clinically indicated. Benzodiazepines are commonly used as the primary treatment for severe agitation, anxiety and seizures, whereas antipsychotic agents may be considered if severe or persistent psychotic symptoms occur. Other interventions may include antiemetics to treat nausea and vomiting, correcting electrolyte disturbances and managing complications such as hyperthermia, rhabdomyolysis or acute kidney injury. In cases of severe intoxication involving respiratory depression, impaired consciousness, uncontrolled agitation, or seizures, airway protection and ventilatory support may be necessary. Therefore, treatment should be tailored to the individual according to their clinical presentation and the severity of their intoxication [14,15].

Unfortunately, the pharmacology and toxicology of SCRAs remain poorly understood, primarily due to the limited availability of studies involving human biological fluids, largely a result of ethical constraints. However, scientific research has shed light on the function action of the endocannabinoid system. This system consists mainly of cannabinoid receptors, to which cannabinoid compounds bind with varying affinities, thereby triggering a range of physiological effects and responses [2,9]. Cannabinoid receptors are G protein-coupled receptors (GPCRs) characterized by seven transmembrane domains. These receptors are associated with heterotrimeric G proteins composed of three subunits: α, β, and γ. Upon activation, cannabinoid receptors induce the dissociation of G protein into the α and βγ subunits. These subunits mediate intracellular signaling pathways by interacting with various second messengers and effector proteins. The two primary cannabinoid receptors identified are cannabinoid receptors type 1 (CB1R) and type 2 (CB2R) [9,16].

CB1R is transcribed by the cnr1 gene and is composed of 472 amino acids in humans [17]. It is involved in the regulation of several physiological and metabolic processes and is responsible for most of the pharmacological effects, including the mediation of psychoactive and neurobehavioral effects. This receptor is mainly found in the central nervous system (CNS), being predominantly abundant in different parts of the brain, such as the prefrontal cortex, which participates in cognitive and emotional processes; the cerebellum and basal ganglia, which participate in motor coordination; and the hippocampus, which is associated with memory. In addition, it is also found in the peripheral nervous system (PNS), where it is most expressed in sympathetic nerve terminals and dermic nerve endings of primary sensory neurons, where it participates in the regulation of nociception from afferent nerve fibers [17,18]. CB1Rs are also present in peripheral tissues such as the liver and pancreatic islets, where they play important roles in regulating glucose homeostasis, lipid metabolism, and insulin secretion [17].

CB2R is transcribed by the cnr2 gene and consists of 360 amino acids in humans [18]. This receptor is involved in the regulation of various protein kinase cascades involved in cell proliferation and survival and can mediate many physiological processes, such as cell differentiation, bone remodeling, and immune responses, and influence the body’s resistance to allergies and oncological and infectious diseases. CB2Rs are in macrophages in the spleen and on the surfaces of immune system cells, and they can also be found in the brain with a postsynaptic location in neurons, glia, and endothelial brain cells [17,18].

**Figure 3:**
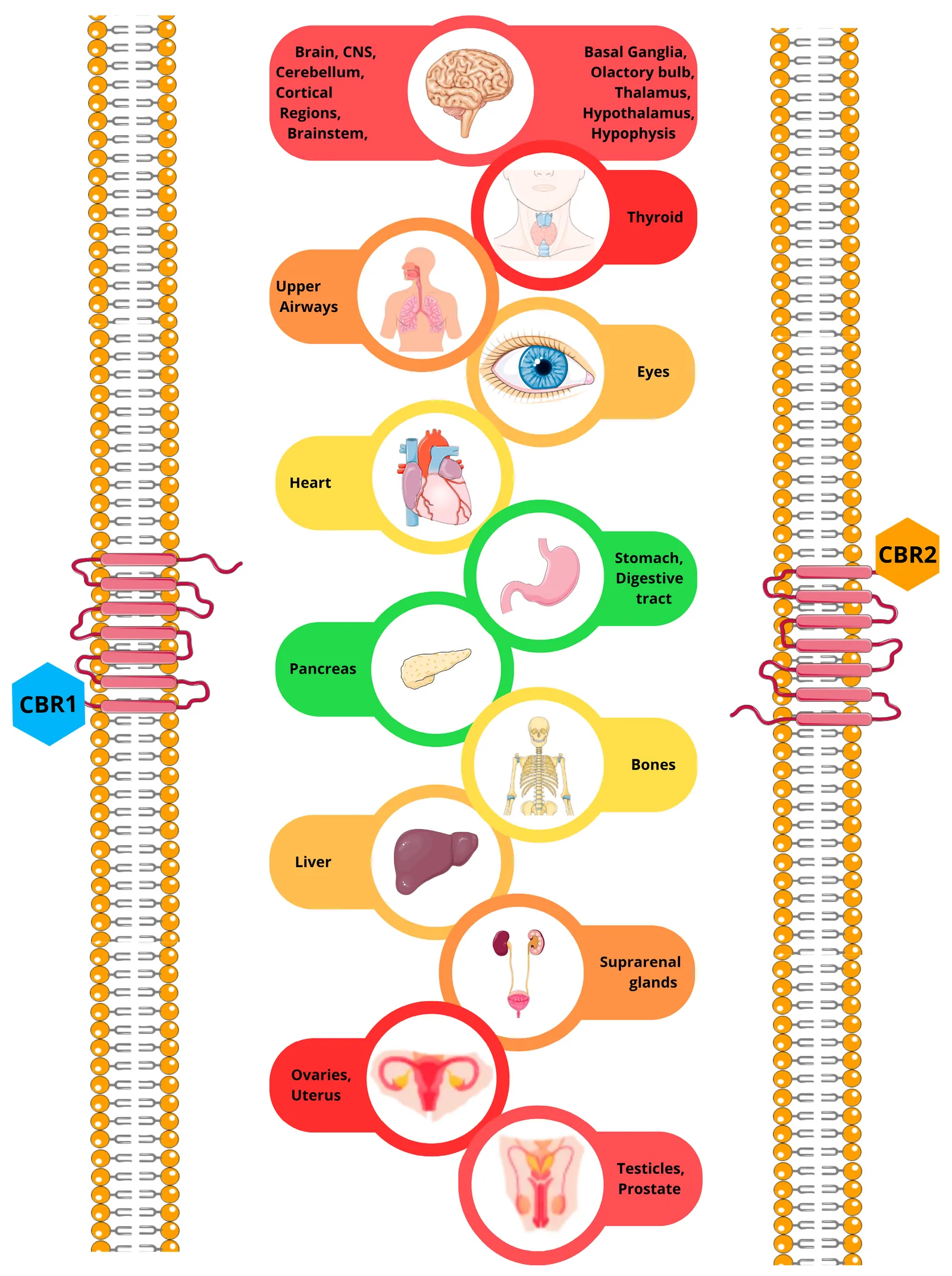
Representation of CBR1 and CBR2 in human organisms. Created using Canva (https://www.canva.com/).

Both receptors are also found in other organs and systems, such as the reproductive system and gastrointestinal tract, as well as in the heart, the lungs, bone, the vascular endothelium, the liver, adipose tissue and skeletal muscle [17]. The locations of CB1R and CB2R are shown in Figure 3.

As previously described, there are three main classes of cannabinoid compounds that act as agonists of the cannabinoid receptors: endocannabinoids, phytocannabinoids, and synthetic cannabinoids. In this review, we will focus specifically on two synthetic cannabinoids: ADB-BUTINACA and 5F-ADB.

ADB-BUTINACA (N-(1-amino-3,3-dimethyl-1-oxobutan-2-yl)-1-butyl-1H-indazole-3-carboxamide) is a new entry on the synthetic cannabinoid list. It is an agonist that belongs to the indazole-3-carboxamide group and was first identified in 2019 in Japan. The substance was regarded as a new psychoactive compound and came after the international control of its structural relative ADB-FUBINACA [19,20]. The main action of ADB-BUTINACA is that it binds and activates the CB1 and CB2 receptors with high potency, being even more efficacious than Δ^9^-THC. The literature suggests very strong psychoactive effects, in addition to acute intoxication that could involve agitation, loss of consciousness, respiratory depression, and convulsions as symptoms [20,21,22]. The compound’s high potency and rapid onset of action have made it a very dangerous substance, as its use has been linked to several deaths.

5F-ADB, which is also called 5F-MDMB-PINACA, is a derivative of indazole-3-carboxamide, a methylated compound that has a fluorinated pentyl side chain, thereby increasing lipophilicity and enhancing permeability across the blood–brain barrier. This, in turn, increases its binding affinity and potency to CB1R. Initially identified in 2014, 5F-ADB soon became one of the most widespread synthetic cannabinoids among seized products all over the world [19,23]. It is currently one of the most toxic synthetic cannabinoid receptor agonists, judging by its strong involvement in fatal intoxication cases and serious neurotoxic effects even on humans and animals chronically exposed at low levels. Among the negative consequences, loss of consciousness, respiratory failure, seizures, and cardiac arrest could be mentioned. The very high potency and unpredicted pharmacological profile of 5F-ADB make it a serious public health issue among the new psychoactive substances [19].

In other vivo studies performed in rodents, 5F-ADB has been shown to suppress the release of key neurotransmitters, such as glutamate and γ-aminobutyric acid (GABA), further indicating its neurotoxic potential [14].

## 4. Toxicokinetics and Toxicometabolomics

The most common route of synthetic cannabinoid consumption is inhalation, which leads to rapid absorption through the upper respiratory tract. This mode of administration results in swift entry into the bloodstream, systemic distribution and a fast onset of psychoactive and physiological effects. SCRAs can also be ingested orally, often as an infusion; however, this route results in lower and more variable bioavailability. Absorption of cannabinoids following oral administration depends on several interrelated factors, including one’s gastric pH, gastrointestinal motility, digestive activity, food intake, intestinal permeability, and presystemic metabolism prior to its entry into the bloodstream. This first-pass effect—the metabolism of xenobiotics before they reach the systemic circulation—occurs predominantly in the liver, although substantial biotransformation can also take place in the intestinal epithelium, where drug-metabolizing enzymes and transport proteins strongly influence oral drug absorption; other extrahepatic tissues, such as the lungs, may also contribute to the metabolism of specific compounds [14]. In the case of synthetic cannabinoids, this presystemic metabolism is mediated primarily by cytochrome P450 isoforms (notably CYP3A4), UGTs, and carboxylesterases (CESs) [24]. While CYP enzymes are responsible for oxidative biotransformation, carboxylesterase 1 (CES1) plays a particularly important role in hydrolyzing ester-containing synthetic cannabinoids, generating carboxylic acid metabolites that subsequently undergo phase II conjugation [25,26]. Collectively, these processes reduce oral bioavailability and contribute to the delayed and highly variable onset of effects characteristic of orally administered synthetic cannabinoids [9,14]. The contribution of the gut microbiota to this metabolic landscape remains poorly characterized; nonetheless, growing evidence suggests that intestinal microbial communities may indirectly modulate xenobiotic disposition by influencing intestinal metabolism and barrier function [27].

**Figure 4:**
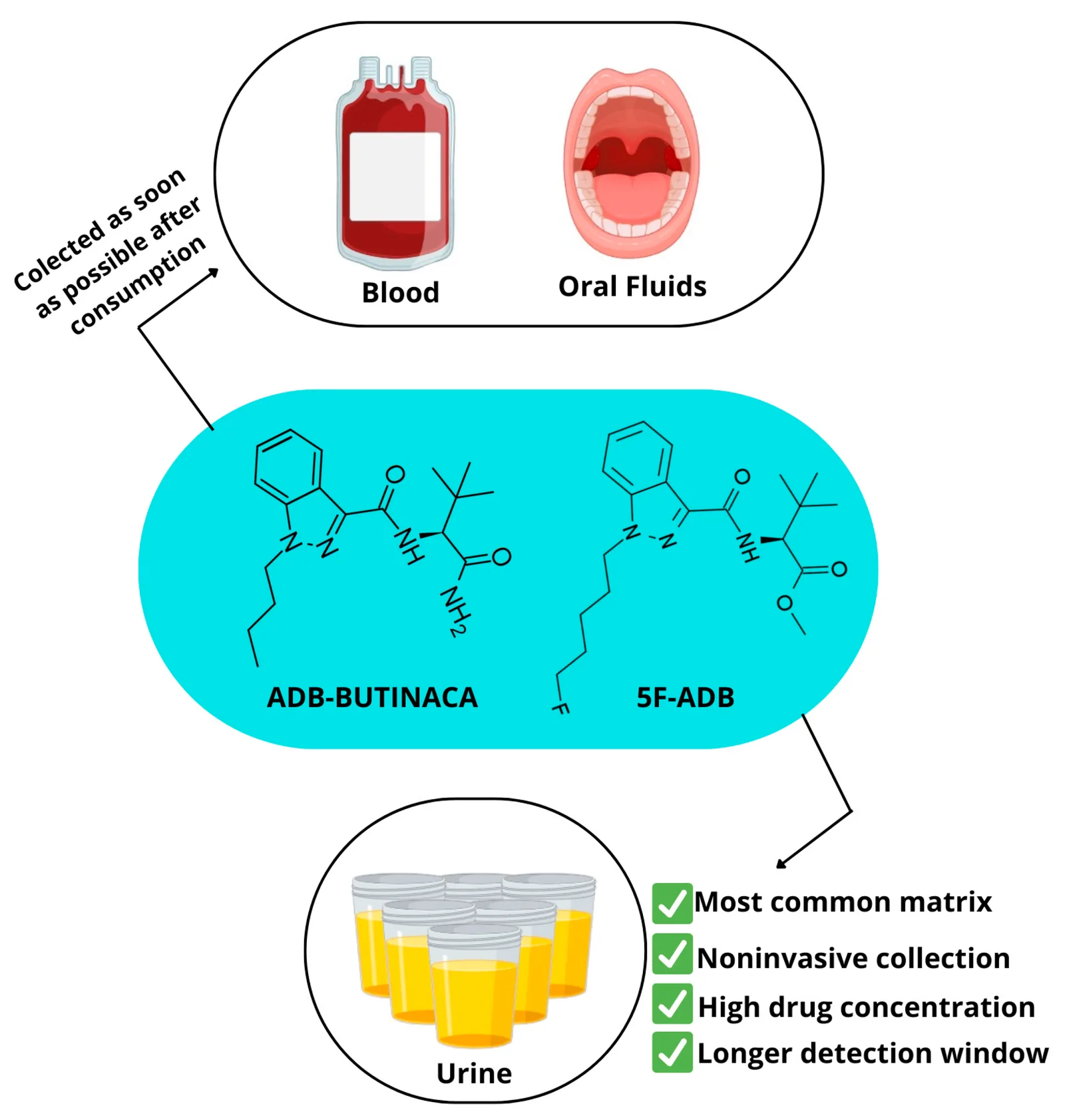
Biological samples wherein it is possible to find evidence of SCRA consumption. Created using Canva (https://www.canva.com/).

SCRAs can be detected in human blood and oral fluid when samples are collected shortly after consumption. Nonetheless, urine remains the most widely used biological matrix for drug testing due to its non-invasive collection, typically higher concentrations of drug metabolites, and longer detection window compared with other sample types (Figure 4).

In blood, parent compounds can still be detected; however, the detection window is shorter than that in urine. Because the concentrations of exogenous substances are often very low and have a very short half-life, highly sensitive analytical techniques are required—typically 10 to 100 times more sensitive than those used for urine analysis. Blood testing also presents several disadvantages, including the invasiveness of the sampling procedure, the requirement for trained personnel to collect the samples, and the high cost of analysis [28,29].

Urine is one of the most analyzed matrices in forensic toxicology. Its main advantages include the detection of most drug metabolites, which allows for a longer detection window compared to other body fluids. Additionally, urine collection is non-invasive and typically yields higher analytical concentrations, making it suitable for drug testing [28,29,30]. However, a notable limitation is that parent compounds, such as the synthetic cannabinoids ADB-BUTINACA and 5F-ADB, are generally not detected. Instead, only their metabolites are found, and the identification of these compounds can be challenging because many of their metabolites are not well characterized [9,14].

Oral fluid is another biological matrix increasingly used for the detection of SCRAs in forensic and clinical toxicology. Compared with blood, it offers a rapid, non-invasive, and easy-to-collect alternative that does not require specialized medical personnel. However, the interpretation of oral fluid results requires caution, as parent synthetic cannabinoids detected shortly after smoking or vaping may reflect residual contamination of the oral cavity rather than systemic circulation. Consequently, oral fluid concentrations may not accurately reflect the actual level of exposure, particularly when samples are collected soon after drug use. Additionally, oral fluid samples are typically smaller in volume than urine samples, and the corresponding drug concentrations tend to be lower, further limiting analytical sensitivity. Physiological factors, such as drug-induced hyposalivation, may further complicate sample collection and testing [28,29,31]. The main characteristics, advantages, limitations, and detection windows of blood, urine, and oral fluid for SCRA analysis are summarized in Table 1.

**Figure 5:**
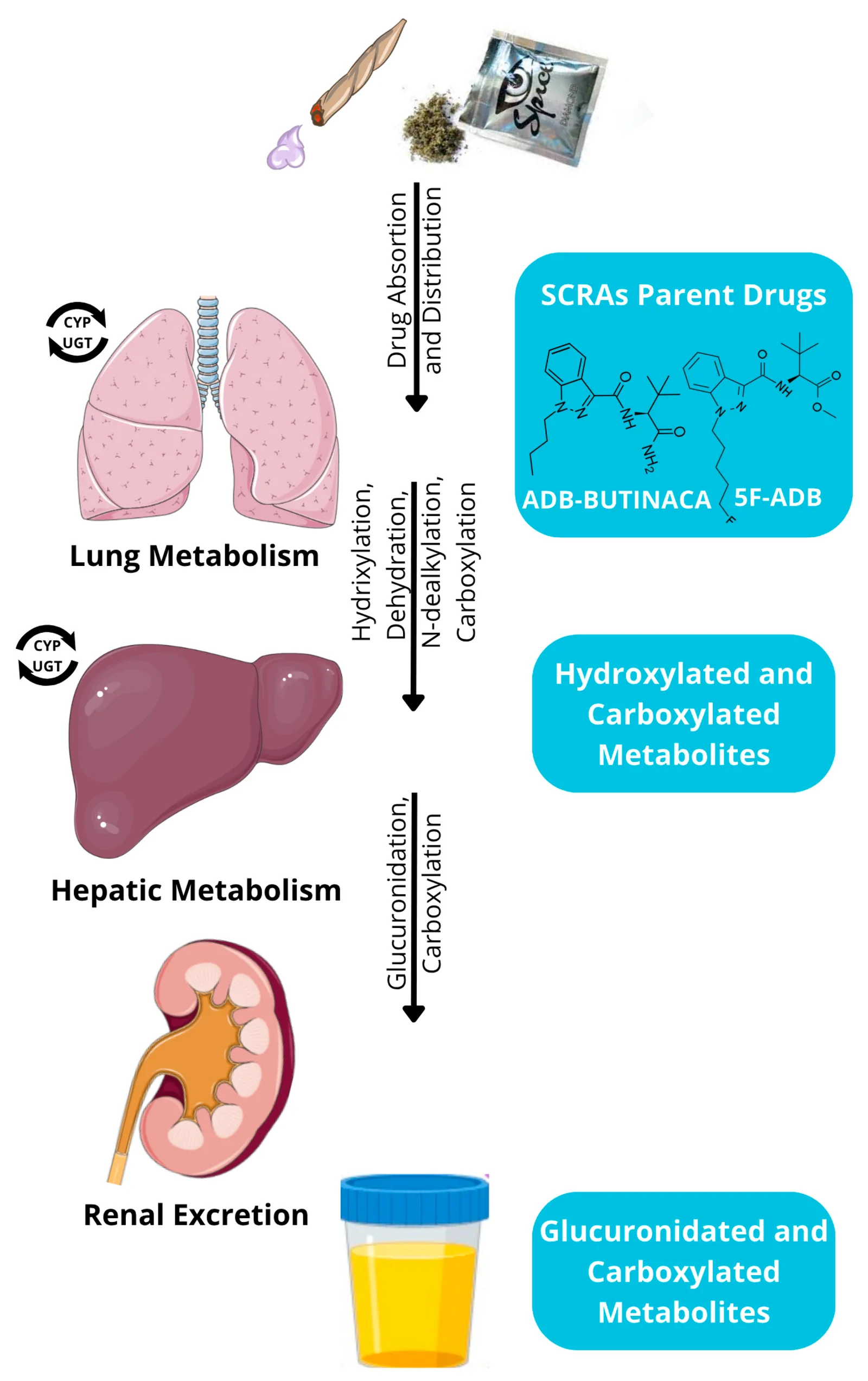
Representation of absorption, distribution, metabolism, and excretion of SCRAs. Created using Canva (https://www.canva.com/).

As previously mentioned, SCRAs are lipid-soluble and non-polar compounds, and their high lipophilicity makes them prone to both phase I and phase II biotransformation reactions. The phase I reaction is typically characterized by alkyl and aromatic oxidation, mainly catalyzed by cytochrome P450 (CYP) enzymes, but it can also involve other enzymes, such as flavin-containing monooxygenases, monoamine oxidases, cyclooxygenases, dihydrodiol dehydrogenases, NAD(P)H quinone oxidoreductases, alcohol dehydrogenases, and aldehyde dehydrogenases. Phase II, in turn, is characterized by conjugation processes, most commonly glucuronidation and sulfation catalyzed by enzymes such as UGTs, glutathione S-transferases (GSTs), sulfotransferases (SULTs), N-acetyltransferases (NATs), and epoxide hydrolases (EHs). The latter play a key role in converting epoxides into dihydrodiols, primarily acting on the CYP-generated phase I metabolites. Ultimately, these transformations facilitate elimination, predominantly via renal excretion, as illustrated in Figure 5. Oxidative metabolism results primarily in the formation of mono-, di-, and trihydroxylated, carboxylated, and N-dealkylated metabolites. Aromatic SCRAs can produce mono-, di- and trihydroxylated metabolites. At the structural level, hydroxylation can occur on the aliphatic chain, in the naphthalene or indole ring systems, or in the substituted aromatic rings. Following secondary metabolism to carboxylic acids, these hydroxylated compounds may undergo conjugation with glucuronic acid [9,14,33].

The first reports on SCRA metabolism appeared in the early 2000s. Since then, the popularity of SCRAs has led to the study of a wide variety of these compounds by researchers around the world. Among them, ADB-BUTINACA and 5F-ADB (5F-MDMB-PINACA) have attracted considerable attention due to their high potency and frequent detection in seized products and biological samples linked to acute intoxication. ADB-BUTINACA, an indazole-based cannabinoid, known for its high potency and rapid metabolism, was reported by Sia et al. (2021) [23], who employed human liver microsomes, recombinant CYP enzymes, and authentic human urine samples. The authors identified fifteen phase I metabolites, primarily resulting from monohydroxylation.

N dealkylation, dihydrodiol formation, and oxidative deamination. The major metabolites were monohydroxylated derivatives, which demonstrated greater metabolic stability than the parent compound. Based on their relative abundance and detectability, four urinary biomarkers were proposed as forensic targets, all of them oxidative deamination products [21].

Kronstrand et al. (2022) [22] later confirmed this data by analyzing post-mortem samples, including urine, blood, kidney, and liver samples from individuals with suspected ADB-BUTINACA consumption. They verified the presence of the previously described metabolites and identified CYP2C19, CYP3A4, and CYP3A5 as the primary isoenzymes responsible for ADB-BUTINACA biotransformation. This dual confirmation, from both in vitro and in vivo data, underscores the analytical importance of these metabolites and thus supports their inclusion in toxicological screening panels [20].

5F-ADB, also known as 5F-MDMB-PINACA, is a methylated indazole 3-carboxamide derivative with a fluorinated pentyl side chain, which enhances lipophilicity and permeability across the blood–brain barrier, thereby also contributing to its high potency at CB1R. 5F-ADB has been termed one of the most toxic SCRAs, given how it has been involved in several fatal intoxication cases and neurotoxic effects even at low levels of exposure [19,23]. Recent studies with human liver microsomes and liquid chromatography–high-resolution mass spectrometry (LC–HRMS) have established that hydrolysis of esters is the major pathway for its metabolism, while oxidative defluorination, hydroxylation, and glucuronidation are minor ones; however, comprehensive metabolic profiling remains limited [19].

The toxicity of the compound has also been demonstrated by clinical reports. One of these reports includes 16-year-old teenagers who developed seizure-like symptoms after accidentally inhaling 5F-ADB through e-cigarette vaping. Analytical confirmation was obtained by analyzing biological fluids by liquid chromatography tandem mass spectrometry (LC-MS/MS), supporting a diagnosis of acute cannabinoid intoxication [23]. Due to their extreme potency and the long half-lives of their metabolites, with the parent compound mostly absent in various biological matrices, forensic detection of ADB-BUTINACA and 5F-ADB relies primarily on metabolic fingerprinting rather than parent drug identification. Together, these SCs exemplify the analytical and toxicological challenges posed by newer generations of synthetic cannabinoids. Their rapid metabolism, structural diversity, and high receptor-binding potency necessitate the use of advanced bioanalytical techniques and accurate metabolite characterization, as well as the constant updating of screening libraries to ensure both public safety and the integrity of evidence.

## 5. In Vitro Models

In vitro models are important for understanding the metabolism, biotransformation, and toxic effects of synthetic cannabinoids (SCs). Unlike in vivo studies, these models provide a more affordable, reproducible, and ethical method for exploring phase I and phase II metabolic pathways, identifying metabolic markers, assessing enzyme activity and drug interactions, and predicting toxic effects. When combined with advanced analytical tools like LC-MS/MS and high-resolution mass spectrometry (HRMS), these models allow for detailed metabolite profiling. They also help identify new markers that are important for forensic and clinical toxicology [22,32,34].

The main in vitro models used to study SCRA metabolism are pooled human liver microsomes (pHLMs), hepatocyte cultures, recombinant enzyme systems, and precision-cut liver slices. These models reflect different parts of liver metabolism and offer useful information on the metabolic pathways involved in SCRA biotransformation. The main characteristics, advantages, and limitations of these in vitro models are summarized in Table 2. Among the available assays, the following are particularly suited to investigating the metabolic pathways of SCs:

(i) Pooled human liver microsomes (pHLMs): pHLMs are among the most widely used in vitro models for studying phase I metabolism driven by cytochrome P450 (CYP) enzymes. pHLMs help identify hydroxylated, carboxylated, and other oxidative metabolites. This makes them especially useful for forensic toxicology and discovering biomarkers. For example, pHLMs have been successfully used to characterize the metabolism of synthetic cannabinoids like PX-1 and PX-2. This research has revealed that CYP2E1 is one of the enzymes involved in their transformation [35].
(ii) HepG2 cell-based assays: HepG2 cells are commonly used to study metabolic pathways and the toxic effects of SCs. Although their CYP enzyme activity is lower than that of primary hepatocytes, they are a useful model for studying phase I and phase II metabolism alongside toxicity. This model has been employed to characterize compounds such as CUMYL-THPINACA and ADAMANTYL-THPINACA and to enhance our understanding of their toxicokinetics [34,36].
(iii) Primary hepatocyte cultures: Primary hepatocytes are one of the most physiologically relevant in vitro models since they retain phase I and phase II metabolic activities. They are therefore particularly suitable for the investigation of glucuronidation, sulfation and other conjugation reactions involved in SCRA metabolism. Human cryopreserved hepatocytes have been successfully used for the characterization of synthetic cannabinoid metabolism, supporting metabolite identification and biomarker discovery in forensic and clinical toxicology [12,32].
(iv) Recombinant enzyme systems: Recombinant human CYP enzymes provide a focused approach for identifying specific isoenzymes involved in SCRA metabolism and for evaluating differences in metabolic capacities between individuals. These systems have been shown to be involved in the metabolism of different SCRAs by CYP2E1 and other CYP isoforms. These systems provide important information on the biotransformation pathways specific to different enzymes and the potential metabolic variability between individuals [35].
(v) Precision-cut liver slice models: These models preserve the three-dimensional architecture and functional enzymatic activity of hepatic tissue, providing a model that closely resembles in vivo conditions. While their use in SCRA research is still in its early stages, they show great promise in the investigation of xenobiotic metabolism and could be a valuable tool in future studies on the metabolic fate of emerging synthetic cannabinoids [37].

## 6. Analytical Approaches

The analysis of drugs and other xenobiotics in complex biological matrices, such as blood, saliva, and urine, is challenging because of the highly complex composition of these biological fluids, which contain a wide range of proteins, salts, lipids, acids, bases, and other endogenous components that may interfere with analyte detection and quantification. Sample preparation is therefore a critical step in the analytical workflow, as it converts complex biological matrices into extracts suitable for reliable instrumental analysis while minimizing matrix-related interferences. Among the available approaches, extraction techniques play a particularly important role by isolating and concentrating the target analytes, removing interfering matrix components, and, consequently, improving the detectability of low-abundance compounds. Effective sample preparation can therefore contribute to enhanced analytical sensitivity, selectivity, precision, and accuracy while also improving the overall robustness, reproducibility, and reliability of the analytical method [14,38].

Conventional sample preparation methodologies include liquid–liquid extraction (LLE), solid-phase extraction (SPE), and protein precipitation (PP), all of which remain widely employed in forensic and clinical toxicology. More recently, advanced microextraction techniques have gained prominence, offering distinct advantages such as reduced sample and solvent consumption, improved chemical specificity, and enhanced selectivity over classical approaches. The selection of an appropriate sample preparation strategy must be meticulously tailored to the specific analytical context, considering the complexity of the biological matrix, the necessity for chromatographic separation, the analyte polarity, and the performance requirements of the intended application.

The wide variety of cannabinoid receptor agonists, or SCRAs, makes it hard to detect them accurately and precisely. Each time the core shape, the linker, the group or the final substituent is changed, a new compound appears with different physical and chemical traits, different ways of being broken down by the body and different signals in tests. In addition, SCRAs that look alike can produce similar breakdown products, which makes it tough to find a urine marker that points to one parent SCRA. Because of this, a test must use a chromatography method that can separate the SCRAs well and a mass spectrometer that can pick up the SCRAs with sensitivity and clear identification. The test must also be updated all the time to include SCRAs and their unique breakdown products. A broad screening test covers more of the fast-moving SCRA market, while a specific quantitative test gives sensitivity and more reliable numbers for each SCRA [9,29,38].

### 6.1. Sample Preparation

#### 6.1.1. Protein Precipitation (PP)

Protein precipitation (PP) is a widely used and straightforward sample preparation technique for the treatment of protein-rich biological matrices, including whole blood, serum, and plasma. The procedure involves the addition of a protein-precipitating agent, which reduces protein solubility and induces protein aggregation and precipitation. The resulting precipitated proteins are subsequently removed by centrifugation or filtration, yielding a protein-depleted supernatant suitable for instrumental analysis. In sample clean-up procedures, acetonitrile, perchloric acid, and trichloroacetic acid are among the most employed precipitating agents. PP offers several advantages, including simplicity, rapid processing, low cost, and minimal solvent consumption, making it particularly attractive for high-throughput applications. However, its main limitation is the limited selectivity of the clean-up process, as endogenous matrix components and other potentially interfering substances may remain in the extract. Consequently, PP alone may provide insufficient matrix removal, potentially leading to ion suppression or enhancement, reduced analytical selectivity, and decreased accuracy and sensitivity, particularly in highly complex biological matrices [39,40,41].

#### 6.1.2. Liquid–Liquid Extraction (LLE)

Liquid–liquid extraction (LLE) is a widely used sample preparation technique for the extraction of organic compounds from biological matrices. The method is based on the partitioning of analytes between two immiscible phases, typically an aqueous biological matrix and an organic solvent, according to their physicochemical properties, particularly their polarity and solubility. Non-polar or lipophilic analytes, such as synthetic cannabinoids, preferentially partition into the organic phase, while more polar matrix components remain predominantly in the aqueous phase. Commonly used organic solvents include n-heptane, n-hexane, ethyl acetate, dichloromethane, and methyl tert-butyl ether (MTBE), with solvent selection depending on the chemical characteristics of the target analytes and the biological matrix. LLE offers several advantages, including a relatively simple procedure, good extraction efficiency for lipophilic compounds, and the effective removal of a substantial proportion of interfering matrix components. However, the technique also presents important limitations, including the need for relatively large volumes of organic solvents, multiple extraction and transfer steps, potential analyte loss during phase separation, and limited automation compared with modern miniaturized sample preparation approaches. In addition, the organic extract generally requires a subsequent evaporation or drying step before instrumental analysis, making the overall procedure relatively time-consuming and increasing the risk of contamination and variability [38,41,42]. These limitations have encouraged the development and application of solid-phase extraction (SPE) as an alternative approach for improving sample clean-up, reducing solvent consumption, and facilitating more efficient and selective sample preparation.

#### 6.1.3. Solid-Phase Extraction (SPE)

The solid-phase extraction (SPE) technique is frequently used for sample cleaning and preparation, where the analytes are captured on a solid sorbent and subsequently desorbed. In contrast to liquid–liquid extraction (LLE), the separation process occurs between a solid phase and liquid sample. SPE presents several benefits, such as the generation of very pure extracts, high selectivity amongst the different compounds present, the elimination of emulsion formation, the reduced usage of solvents, and the easy automation of the process. Bear in mind though that it might become a pricey and lengthy procedure, and the matrix effects might be caused by the insufficient retention of the endogenous compounds. Its flexibility is due to the great variety of sorbents available, which allows for patently different optimizations of protocols. The SPE method typically consists of a sequence of four operations: conditioning, sample loading, washing, and, finally, elution [43,44].

#### 6.1.4. QuEChERS

QuEChERS (Quick, Easy, Cheap, Effective, Rugged and Safe) is an extraction method initially developed for pesticide residue analysis and later adapted for different analytical applications. It consists of mainly two stages: extraction of analytes and partitioning, in addition to clean-up done by means of dispersive solid-phase extraction (d-SPE). QuEChERS is characterized by its simplicity, versatility, and cost-effectiveness. However, it may also be seen as a labor-intensive, time-consuming process that requires relatively large sample volumes and the use of toxic solvents. In such cases, protocol modifications are frequently required to prevent the degradation of target compounds and optimize the extraction of pH-sensitive analytes [45,46].

#### 6.1.5. Solid-Phase Microextraction (SPME)

Solid-phase microextraction (SPME) is a novel and efficient sorption-based sample preparation technique that is a simple, economical, and versatile alternative to traditional chromatographic methods. It gives the benefits of easy automation, the use of less solvent, and the ability to work in the field. In SPME, the analytes are first exposed to the headspace and then later taken out of a polymer-coated fused silica fiber by the DI-SPME or HS-SPME techniques depending on whether they are non-volatile or volatile compounds, respectively. The solvent-free and eco-friendly method is a perfect candidate for gas chromatography coupling. Notwithstanding, SPME is hampered by the fragility of the fibers and the narrow selection of coatings available [47,48].

#### 6.1.6. Miniaturized Solid-Phase Extraction (μ-SPE)

Miniaturized solid-phase extraction (μ-SPE) is a micro-scale version of conventional SPE that packs up pipette tips with tiny amounts of sorbent. Unlike the standard SPE that requires vacuums, μ-SPE uses micropipettes to load, clean, and elute the sample. Commercial or lab-made monolithic sorbents are the usual ones in this process. μ-SPE has some advantages over SPME in suspensions and semi-solid samples, as it eliminates particle contamination, minimizes solvent consumption, and enables the use of one device 20 times. It is quicker and cheaper and does not have problems such as fiber breakage or analyte carryover. Researchers are still looking at the interactions between sorbent and sample to find better supportive materials for the tests [38,49].

#### 6.1.7. Microextraction by Packed Sorbent (MEPS)

Microextraction by packed sorbent (MEPS) is a small-scale method which not only reduces the use of substances and therefore the environmental footprint but also offers faster and simpler sample processing compared to conventional solid-phase extraction (SPE). MEPS uses a small sample size but still achieves a high extraction efficiency measure. The device packs into itself all the processes of extraction, preconcentration and clean-up and is thus perfect for coupling with liquid or gas chromatography for complete automation. Offering a similar performance to that of classical SPE, MEPS is selective, adaptable, and ideal for both analytical and bioanalytical works, and it has the same workflow, namely, conditioning, loading, washing, and elution, just with lower volumes and more flexibility of operation [50,51,52].

#### 6.1.8. μSPEed Extraction

μSPEed extraction is a high-level MEPS process with substantial design enhancements. It operates with cartridges that have a one-way check valve actuated by pressure and a very low dead volume, which guarantees that the sample flows in only one direction through the sorbent bed. This arrangement permits the sample to be drawn in by vacuum only, which results in lower flow resistance and, consequently, better extraction. In addition, μSPEed uses very small sorbent particles (≤3 μm) in a small cartridge, providing better performance and accuracy than traditional MEPS, which uses larger (≈50 μm) particles [53,54].

### 6.2. Analytic Techniques

Colorimetric assays are commonly used as presumptive tests for preliminary screening; however, they generally lack the sensitivity and specificity required for the reliable detection of SCs. Moreover, these assays frequently respond to structurally related compounds, leading to potential misidentification. In routine forensic immunoassays, cross-reactivity has resulted in several cases of structural similarities among produced false-positive or false-negative outcomes [55,56].

The situation becomes even more challenging when SCs are chemically modified, as such alterations hinder the generation of specific antibodies needed for accurate immunoassay detection. Given these limitations, advanced analytical techniques, such as infrared spectroscopy, gas chromatography–mass spectrometry (GC–MS), and nuclear magnetic resonance (NMR) spectroscopy, are essential for the definitive identification of SCs in forensic and toxicological investigations.

#### 6.2.1. Colorimetric Methods

Colorimetric techniques have long been used for the rapid and low-cost detection of substances such as narcotics and explosives. They are based on chemical reactions that produce visible color changes measurable by spectrophotometry. Traditionally applied to drugs like marijuana, cocaine, and heroin, these methods have been adapted for detecting synthetic cannabinoids (SCs). However, the structural diversity of SCs complicates standard testing. Modified reagents such as Fast Blue B and Ehrlich’s, along with oxidative and acid-based reactions, have shown promising results. Recent innovations include nanomaterial-enhanced and paper-based sensors for improved sensitivity and field portability. Despite their simplicity and speed, colorimetric methods suffer from false positives and poor quantification accuracy. To overcome these issues, researchers are integrating digital analysis and developing more selective reagents, aiming to keep detection methods aligned with the rapid evolution of SCs [55,56,57].

#### 6.2.2. Immunoassays

Immunoassays are widely used for screening in clinical and forensic toxicology because they allow for the rapid analysis of biological samples. They are based on the selective interaction between an antigen and its corresponding antibody and are typically performed using competitive assay formats, in which labeled and unlabeled analytes compete for antibody-binding sites. Although immunoassays are rapid, simple to perform, and suitable for high-throughput screening, their performance may be affected by cross-reactivity, which can result in false-positive or false-negative results. Consequently, confirmation by chromatographic–mass spectrometric methods are generally required [14].

Several commercial immunoassays have been developed to screen for SCRAs and their metabolites. These include forensic ELISA kits that target JWH-018-, JWH-250- and UR-144/XLR-11-related compounds, as well as HEIAs (homogeneous enzyme immunoassays) that have been developed for different groups of SCRAs. Competitive ELISAs are based on competition between the analyte present in the sample and enzyme-labeled drug conjugate for a limited number of antibody-binding sites. Following incubation and washing, adding a chromogenic substrate such as 3,3′,5,5′-tetramethylbenzidine (TMB) produces an enzyme-dependent color reaction. The intensity of the signal is inversely related to the amount of target analyte present in the sample. HEIAs also rely on competitive antigen–antibody interactions, but they generate a homogeneous, enzyme-mediated signal without the need for separation or washing steps. This facilitates the rapid, automated screening of biological samples [58,59].

Despite being rapid and suitable for high-throughput screening, commercial SCRA immunoassays have significant limitations. Their analytical coverage is determined by antibody cross-reactivity; therefore, structurally related SCRAs and metabolites may produce markedly different responses. A recent evaluation of Immunoanalysis Synthetic Cannabinoid HEIA assays revealed significant differences in the cross-reactivities among cannabinoid analogs and metabolites, depending on the compound. This illustrates that newly emerging or structurally modified SCRAs may not be adequately detected by assays developed against earlier compounds. Consequently, a negative immunoassay result cannot unequivocally exclude exposure to an SCRA when the compound has limited or unknown cross-reactivity with the employed antibody. These assays are therefore most appropriate as rapid preliminary screening tools, particularly for urine. However, positive or clinically or forensically relevant findings should be confirmed using more selective analytical techniques, such as LC-MS/MS or LC-HRMS. The continuous evaluation and updating of assay cross-reactivity profiles is necessary as new SCRAs emerge [58,59].

#### 6.2.3. Thin-Layer Chromatography (TLC)

Thin-layer chromatography (TLC) is a fast, simple, and inexpensive method for preliminary drug screening. Samples applied to a stationary phase separate in a mobile solvent based on their affinities and are analyzed using Rf values [60]. TLC is versatile, environmentally friendly, and allows for the rapid analysis of multiple samples, but it is less selective than advanced chromatographic techniques, often requiring confirmatory methods like HPLC or mass spectrometry [61,62].

#### 6.2.4. Gas Chromatography–Mass Spectrometry (GC-MS)

Gas chromatography–mass spectrometry (GC-MS) is a highly sensitive and specific method widely used in forensic drug analysis. In GC, vaporized samples are transported through a heated column by an inert gas (mobile phase), separated by the stationary phase, and detected as chromatograms. GC-MS is considered the gold standard for drug testing, enabling identification of unknown substances by comparing mass spectra with library databases. It is especially suited for low-molecular weight and volatile compounds. Limitations include the need for sample volatility, the potential degradation of thermally labile compounds, and the poor response of highly polar molecules, often addressed by derivatization [63,64,65].

#### 6.2.5. Liquid Chromatography (LC)

Liquid chromatography (LC) has become the most applied method for the detection of SRCAs and their metabolites in biological samples. One of the main advantages of LC over gas chromatography (GC) is that it handles the problem of thermal degradation that arises from high injector temperatures. In LC, the separation of the analytes is based on their respective retention times, which are determined by the interaction forces of the analytes with the stationary phase while a liquid mobile phase is flowing. The compounds with weaker interactions are the first ones to elute, and those with stronger interactions remain in the column for a longer period. The invention of conventional LC in the 1960s led to high-performance liquid chromatography (HPLC), which is not only more efficient but also a more automated process. In HPLC, the stationary phase consists of small particles with high surface areas, which are assigned to a column packed under high pressure, and the samples are pushed through the column to attain separation quickly and efficiently [66,67,68].

#### 6.2.6. Nuclear Magnetic Resonance

Nuclear magnetic resonance (NMR) has been a very important method for the identification and structural elucidation of unknown compounds. NMR is a very good complementary method to mass spectrometry (LC-MS or GC-MS) in most cases. The technique identifies the signals produced when the nuclear spins interact with the magnetic field and the radiofrequency radiation applied. NMR plays a crucial role in the rapid and non-destructive characterization of complex materials, enabling the acquisition of high-quality structural information with minimal sample preparation. However, compared with LC–MS and GC–MS, NMR has substantially lower analytical sensitivity, which limits its applicability for the detection and characterization of trace levels of synthetic cannabinoids and their metabolites in biological matrices. Consequently, NMR is primarily employed for structural elucidation, purity assessment, compound identification, and metabolomics, where its high reproducibility and quantitative capabilities are particularly advantageous. Rather than replacing mass spectrometry-based approaches, NMR is best regarded as a complementary analytical technique. Moreover, its integration with chromatographic separation and hyphenated approaches, such as LC–NMR, can further enhance its analytical versatility by combining the separation capabilities of chromatography with the detailed structural information provided by NMR [69,70].

### 6.3. Analytical Method Validation and Performance

Analytical methods designed to find and measure SCRAs in clinical toxicology need full testing to make sure the results are correct and can be used properly. Important things to check include how well the method can tell the SCRA apart from things, how specific it is, the way it measures amounts and how it works over a range, the lowest amount it can find and measure, how accurate and repeatable the results are, how much is recovered during testing, how the sample materials affect the results if there is any leftover from previous tests, whether diluting the sample changes the results, and how stable the SCRA is. When needed, the uncertainty in the measurements should also be looked at. These things are especially important for SCRAs because the amounts in body samples can be very small and there can be differences in how well they are extracted, how they are turned into ions, and how the sample affects them between similar compounds and their breakdown products [29,39,41,43,68,71,72].

The level of validation must match the intended use of the analysis. For screening, it is important to have clear identification criteria and enough selectivity and sensitivity to avoid false-negative and false-positive results. When using confirmatory methods, there is an added need to evaluate the calibration performance, accuracy, precision and measurement uncertainty. In analytical methods targeting SCRAs, it is also necessary to account for differences in the extraction efficiency and analytical response that vary by compound. A performance that is acceptable for one SCRA might not apply to similar analogs or metabolites [71,73,74].

Table 3 summarizes the representative validated analytical methods for determining SCRAs and their metabolites in human biological matrices, highlighting differences in sample preparation, analytical platforms, sensitivity, and quantitative performance.

As shown in Table 3, the analytical performances of methods used to determine SCRAs vary significantly depending on the biological matrix, the target analytes, the sample preparation method, and the instrumental platform. Broad-spectrum screening methods offer coverage of analytes but often lack the sensitivity of more targeted approaches. For example, Shi et al. analyzed 179 SCRA compounds and 80 metabolites at the time in blood and urine using liquid chromatography–high resolution mass spectrometry (LC-HRMS) [68]. They achieved limits of detection (LODs) of 5 ng/mL or lower for more than 80% of the compounds. However, Gaunitz et al. reported much lower LODs—ranging from 0.025 to 0.5 ng/mL—for a targeted set of 61 phase I SCRA metabolites in urine using solid-phase extraction coupled with liquid chromatography–tandem mass spectrometry (SPE-LC-MS/MS) [43]. These results highlight the trade-off between covering analytes and achieving high sensitivity. They also show the need to carefully choose a method that matches the goals of the forensic or clinical application.

The biological matrix is another factor when it comes to analytical performance and how results are interpreted. Whole-blood methods can detect the parent SCRAs, which makes them especially useful in cases of intoxication and in forensic settings. Urinary analysis often depends on metabolites, since these substances undergo extensive biotransformation in the body, leading to very low or even undetectable levels of the original compounds. Different matrices offer different kinds of information. For example, oral fluid sampling is minimally invasive and helps assess recent exposure; however, interpreting oral fluid results can be tricky because of leftover contamination in the mouth from smoking or vaping [29]. Hair sampling, by contrast, offers a longer detection window and allows for the evaluation of past SCRA exposure. This means that LOD and LOQ values should not be judged alone. These values must be considered along with the matrix, the target analyte, the sample volume, the extraction method, and the overall purpose of the analysis [75].

Sample preparation is also a factor in analytical performance. Protein precipitation is a method that helps create rapid high-throughput workflows and allows for the simultaneous determination of large SCRA panels in whole blood. However, protein precipitation does not remove endogenous matrix components, and this can increase matrix effects. A selective method, such as solid-phase extraction (SPE), gives better sample clean-up and can help detect SCRA metabolites in complex biological matrices with higher sensitivity. SPE usually needs extra extraction steps and optimization work [38]. Miniaturized approaches, for example DLLME, can reduce both sample size and solvent use while keeping extraction efficiency. Therefore, no single extraction procedure can be called optimal for all SCRA analyses [38,72]. The choice of a procedure must consider the properties of the target compounds, the complexity of the matrix, the required sensitivity, the number of analytes, and the desired analytical throughput. Improving performance depends on good sample preparation [38].

It is important to note that the lowest reported LOD or LOQ should not be regarded as the sole indicator of superior analytical performance. Direct comparisons between methods must consider differences in biological matrices, sample volumes, extraction procedures, target analytes, calibration strategies and instrumental platforms. Instead, method fitness for purpose should be assessed by considering sensitivity alongside selectivity, accuracy, precision, recovery, matrix effects, robustness, analyte coverage, and the requirements of the intended forensic or clinical application [76].

For forensic laboratories operating within an accreditation framework, method validation must be supported by documented quality control procedures and ongoing performance monitoring. This is particularly important when analytical results may support clinical or judicial decisions, as compliance with internationally recognized laboratory competence and forensic toxicology requirements is essential. Therefore, validation must demonstrate that the method is fit for its intended purpose and that its performance remains consistent during routine use [76,77].

## 7. Biomarkers of Exposure

Biomarkers of exposure are increasingly important for the detection and monitoring of SCRAs in humans. Although SCRAs are used and sold illegally in dark internet markets, their harmful effects through unpredictable pharmacological effects make them a major threat to public health. Consequently, the identification of reliable biomarkers for SCRAs is critical for forensic investigation, clinical toxicology and drug monitoring worldwide [78,79].

SCRAs are rapidly metabolized following administration—whether by injection, smoking, ingestion, or other routes—through phase I or phase II metabolic processes. In this context, a metabolite is a breakdown product that retains structural features linking it to the parent SCRA compound. Unlike THC, which may be detected in its original form and as metabolites, intact SCRAs are rarely found in biological samples due to their rapid metabolic clearance [78,79].

Phase I metabolism primarily involves hydroxylation and carboxylation reactions. In contrast, phase II metabolism consists of conjugation of oxidative by-products, which increases the polarity of the SCRAs, allowing further water solubility and facilitating further metabolism and excretion. Hydroxylated and carboxylated metabolites serve as reliable biomarkers for SCRAs and are commonly detected in urine and blood samples. The structural similarity between the parent compound and these metabolites facilitates their identification through targeted analytical methods [78,79].

The conjugation of SCRA metabolites during phase II metabolism with hydrophilic groups, such as glucuronic acid, enhances their excretion. Glucuronidation increases the water solubility of SCRA metabolites, promoting renal clearance. Therefore, glucuronide conjugates serve as valuable biomarkers for detecting SCRAs in urine, where they remain detectable for longer periods than their free metabolites [78,79].

With the continuous emergence of new SCRA analogs with multiple structures, the proposed methods for biochemical detection need to be enhanced and developed further.

Among the biological matrices, urine is the most suitable for detecting SCRA biomarkers due to the high concentration of metabolites and extended detection window. In contrast, blood samples, which contain lower metabolite levels and provide a shorter detection window, are primarily useful for linking exposure to functional impairment. Rapid drug clearance and low concentrations in saliva limit its utility for SCRA detection, whereas hair offers a valuable alternative for long-term monitoring, providing evidence of SCRA use over weeks to months [12,29,75].

Despite significant technological advances in the analysis of SCRAs, their detection remains a major challenge. The rapid evolution of SCRA compounds driven by manufacturers who continually modify chemical structures to evade legislative control represents the foremost challenge. These frequent and unpredictable structural modifications complicate the identification of universal biomarkers, necessitating the continuous adaptation and refinement of analytical screening methods [12,80,81].

Another major issue is the short detection window of SCRA metabolites, which contrasts sharply with Δ^9^-THC and its metabolites, which persist for extended periods in biological matrices. SCRA metabolites, by comparison, disappear rapidly, making their detection within practical timeframes extremely difficult. Furthermore, the lack of standardized cut-off values and the use of inconsistent detection methods across laboratories hinder the reliable interpretation of SCRA exposure, often rendering results difficult or even impossible to compare [12,81].

One of the major focuses of forensic and clinical toxicologists is the identification of biomarkers of synthetic cannabinoid exposure. Developments in mass spectrometry, metabolomics and bioinformatics will, in general, provide the basis for further improvements in the analysis of SCRA metabolites and, hence, the correct drug-testing procedures, as well as for understanding the metabolic disruption in cells due to SCRA exposure. Instead, future initiatives should focus on the development of broad-spectrum screening, real-time detection methods, and standardized analytical protocols that will overcome the challenge posed by unlimited SCRA evolution [40,67].

## 8. Conclusions

The growing prevalence of SCRAs as novel psychoactive substances poses significant analytical and toxicological challenges in both clinical and forensic contexts. This review provides a comprehensive overview of the analytical characterization of SCRAs, highlighting the crucial role of advanced methodologies in detecting these compounds and their metabolites within complex biological matrices.

Toxicometabolomics has emerged as a powerful strategy to unravel the metabolic fate and toxicological consequences of SCRA exposure, delivering critical insights into the biotransformation pathways of SCRAs and the associated health risks. In vitro assays have greatly advanced our understanding of the pharmacological activity, toxicity, and metabolic pathways of these substances, emphasizing the need for the continuous monitoring and rigorous evaluation of emerging analogs. Effective detection of SCRAs relies on optimized sample preparation and the application of robust analytical platforms, particularly chromatography coupled with mass spectrometry, which enables the accurate identification and quantification of SCRAs and their metabolites in biological matrices. Equally critical is the discovery and validation of reliable biomarkers of exposure, which support both forensic and clinical applications, improving detection strategies, and strengthening comprehensive risk assessment.

The integration of artificial intelligence (AI) and machine learning represents a paradigm shift in toxicological research, particularly given the rapid data processing and advanced pattern recognition required to monitor the dynamic landscape of emerging SCRA variants. AI-driven methodologies have the potential to enhance the detection of novel SCRAs, predict toxicological outcomes, and streamline data interpretation, thereby supporting more informed forensic and clinical decision making.

Given the continual evolution of SCRAs, analytical strategies and toxicological research must adapt in parallel. The effective monitoring and mitigation of public health risks associated with SCRA exposure requires a multidisciplinary approach that integrates forensic science, analytical chemistry, and regulatory oversights. The implementation of high-throughput metabolomics, AI-driven analytics, and novel detection technologies will be crucial to keeping pace with the rapid emergence of new synthetic variants. Strengthening collaboration among researchers, law enforcement, and regulatory agencies is essential to address the complex public health challenges posed by these compounds. This review underscores the urgent need for sustained advancements in analytical and toxicological sciences to improve the detection, monitoring, and understanding of SCRAs, ultimately supporting more effective regulatory measures and public health interventions.

## Figures and Tables

**Table 1:** Comparison of biological matrices for SCRA detection, including detection windows, target analytes, and analytical strengths and limitations.

Matrix	Detection Window	Collection Process	Target Compounds	Advantages	Disadvantages	References
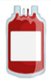 Blood	Hours	Invasive	Parent and drug metabolites	Parent substances and metabolites can be detected	Needs specially trained staff	[29]
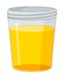 Urine	Hours–days	Non-invasive	Metabolites	Easily accessible and longer detection window	Only metabolites can be detected; need for sample preparation procedures before analysis	[14,30]
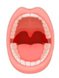 Oral Fluid	Minutes–12 h	Non-invasive	Mainly parent drugs	Easy and quick access and reduced risk of adulteration	Short detection window; only parent substances can be detected	[29,32]

**Table 2:** Comparison of principal in vitro models used to investigate synthetic cannabinoid metabolism.

In Vitro Model	Main Applications	Main Advantages	Main Limitations	References
Pooled human liver microsomes (pHLMs)	Phase I metabolism; CYP-mediated biotransformation	Rapid, reproducible, cost-effective, and suitable for metabolite identification	Limited to microsomal enzymes; lack of cellular environment and phase II metabolism	[32,35]
HepG2 cells	Phase I/II metabolism and cytotoxicity studies	Easy to culture; suitable for metabolism and toxicity assessment	Lower CYP activity than primary hepatocytes	[34,36]
Primary hepatocytes	Phase I and phase II metabolism	Closely resemble human hepatic metabolism; preserve conjugation pathways	Limited availability; donor variability; high cost	[12,32]
Recombinant CYP enzymes	Identification of enzyme-specific metabolic pathways	High specificity for individual CYP isoforms; useful for enzyme phenotyping	Do not reproduce the complexity of hepatic metabolism	[35]
Precision-cut liver slices	Integrated hepatic metabolism	Preserve tissue architecture and cell–cell interactions	Limited viability; low throughput; restricted availability	[37]

**Table 3:** Comparison of analytical methods for the determination of synthetic cannabinoids and their metabolites in biological matrices.

Target Analytes	179 SCRAs + 80 Metabolites	132 SCRAs Within a 182-NPS Panel	61 Phase I Metabolites from 29 SCRAs	JWH-018, JWH-019, JWH-073, JWH-200, JWH-250, AM-694	5F-MDMB-PICA + Five Metabolites
**Matrix**	Human blood and urine	Human whole blood	Human urine	Human oral fluid	Human hair
**Sample Preparation**	ACN protein precipitation	Protein precipitation	SPE	ACN precipitation + DLLME	MeOH extraction
**Analytical Method**	LC-HRMS	LC-MS/MS	LC-MS/MS	LC-MS/MS	UHPLC-MS/MS
**Limit of Detection (LOD)**	≤5 ng/mL for >80%	Analyte-dependent	0.025–0.5 ng/mL	Analyte-dependent	0.5–5 pg/mg
**Limit of Quantification/Lower Limit of Quantification** **(LOQ/LLOQ)**	NR	Analyte-dependent	NR	Analyte-dependent	1–5 pg/mg
**Recovery/Matrix Effect**	Recovery and matrix effects evaluated	Evaluated individually	Recovery 43–97%; matrix effects 81–185%	Extraction recoveries generally >70%	Recovery 61.1–93.3%; matrix effects 19.1–102.6%
**Linear Range/Performance**	Broad screening; 239 targets; 115 authentic forensic samples	0.25–10 ng/mL calibration range for SCRAs	Targeted metabolite screening	Targeted SCRA determination	1–200 pg/mg depending on analyte
**Reference**	[68]	[41]	[43]	[72]	[71]

## Data Availability

No new data were created or analyzed in this study. Data sharing is not applicable to this article.

## Abbreviations

The following abbreviations are used in this manuscript:

AI	Artificial intelligence
CB1R	Cannabinoid receptor type 1
CB2R	Cannabinoid receptor type 2
CBD	Cannabidiol
CESs	Carboxylesterases
CES1	Carboxylesterase 1
CNS	Central nervous system
CYP	Cytochrome P450
DI-SPME	Direct immersion solid-phase microextraction
DLLME	Dispersive liquid–liquid microextraction
d-SPE	Dispersive solid-phase extraction
GC-MS	Gas chromatography–mass spectrometry
GPCRs	G protein-coupled receptors
HPLC	High-performance liquid chromatography
HRMS	High-resolution mass spectrometry
HS-SPME	Headspace solid-phase microextraction
LC	Liquid chromatography
LC-HRMS	Liquid chromatography–high-resolution mass spectrometry
LC-MS	Liquid chromatography–mass spectrometry
LC-MS/MS	Liquid chromatography–tandem mass spectrometry
LLE	Liquid–liquid extraction
LOD	Limit of detection
LOQ	Limit of quantification
MEPS	Microextraction by packed sorbent
MS	Mass spectrometry
MTBE	Methyl tert-butyl ether
NMR	Nuclear magnetic resonance
NPSs	New psychoactive substances
pHLMs	Pooled human liver microsomes
PNS	Peripheral nervous system
PP	Protein precipitation
SCRAs	Synthetic cannabinoid receptor agonists
SPE	Solid-phase extraction
SPME	Solid-phase microextraction
THC	Δ9-tetrahydrocannabinol
TLC	Thin-layer chromatography
UGTs	UDP-glucuronosyltransferases
μ-SPE	Miniaturized solid-phase extraction
